# 1952. Second Time’s the Charm? Low Yield of 3 AFB Smears among Inpatients Evaluated for Pulmonary Tuberculosis

**DOI:** 10.1093/ofid/ofad500.106

**Published:** 2023-11-27

**Authors:** Caitlin Dugdale, Kimon C Zachary, Dustin S McEvoy, Amy Courtney, Rebecca L Craig, Lindsay Germaine, Chloe V Green, David C Hooper, Emily P Hyle, Molly L Paras, Erica S Shenoy

**Affiliations:** Massachusetts General Hospital, Boston, MA; Massachusetts General Hospital, Boston, MA; Mass General Brigham, Ann Arbor, Michigan; Massachusetts General Hospital, Boston, MA; Massachusetts General Hospital, Boston, MA; Mass General Brigham, Ann Arbor, Michigan; Massachusetts General Hospital, Boston, MA; Massachusetts General Hospital, Boston, MA; Massachusetts General Hospital, Boston, MA; Massachusetts General Hospital, Harvard Medical School , Boston, MA; Massachusetts General Hospital, Boston, MA

## Abstract

**Background:**

Among inpatients with suspected tuberculosis (TB), CDC and IDSA guidelines recommend collecting three respiratory specimens 8-24 hours apart for acid-fast bacilli (AFB) smear and mycobacterial culture. However, data supporting this approach are limited, particularly in the era of Xpert MTB/RIF PCR assays (Xpert). Our objective was to estimate the sensitivity of 1, 2, or 3 AFB smears +/- Xpert to detect pulmonary TB in a low incidence setting.

**Methods:**

We conducted a retrospective study of inpatients at a large U.S. academic medical center with mycobacterial culture performed on one or more respiratory specimens July 2016 – December 2022. AFB smear microscopy, Xpert, or both, were performed on each specimen. We evaluated percent positivity on serial AFB smears and on Xpert among patients with confirmed TB, as well as the yield of a third AFB smear among all patients tested.

**Results:**

A total of 7,445 inpatients underwent 15,710 mycobacterial cultures on respiratory specimens, of whom 52 (0.7%) were diagnosed with culture-positive pulmonary TB (Figure 1). Among patients with TB, the first AFB smear was positive in 22/52 (42%) cases and a second AFB smear was positive in 5/24 (21%) cases; there were no cases in which a third AFB smear was positive after two initial negative smears (Table 1). Among 1,523 instances in which a third specimen was obtained among patients with and without TB, the third sample yielded a diagnosis of TB by culture in 1 case. Overall sensitivity to detect a positive AFB smear or Xpert result among patients with TB was equivalent between 2 and 3 AFB smear pathways at 52%. Overall sensitivity to detect TB prior to culture positivity was highest with addition of an Xpert to 2 or 3 AFB smears at 67% (Table 2).

**Figure:**
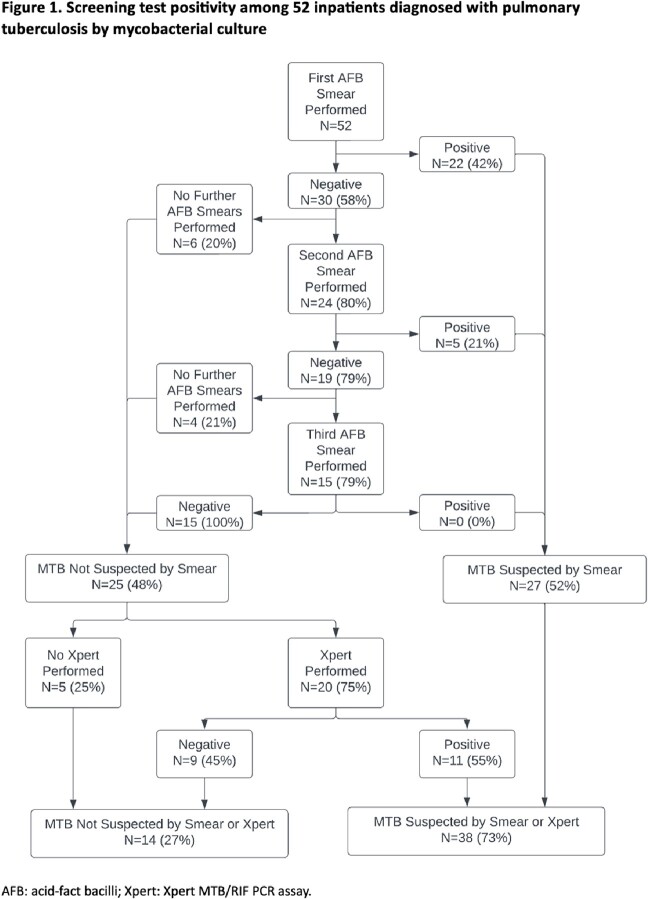

**Figure:**
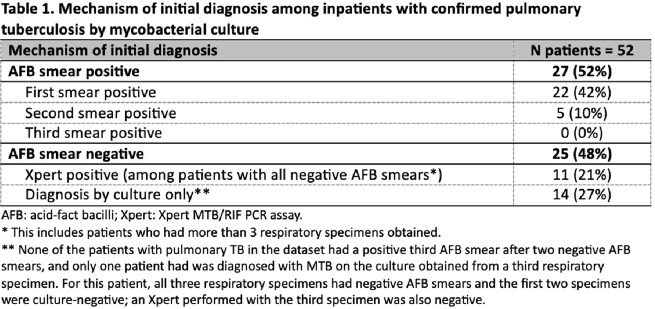

**Figure:**
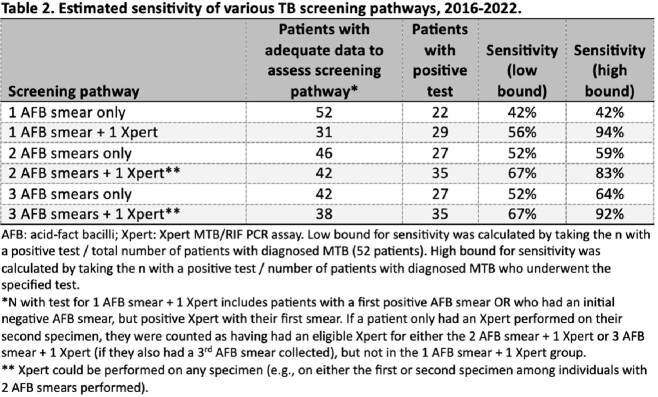

**Conclusion:**

In a low incidence setting, TB screening with 2 AFB smears offers the same diagnostic yield as with 3 AFB smears while potentially reducing laboratory burden and duration of patient isolation. Xpert demonstrated high sensitivity for detection of TB, and it increased sensitivity to detect TB when added to AFB smear-based screening alone. However, a quarter of patients with TB diagnosed by mycobacterial culture were not detected by AFB smear or PCR, highlighting the importance of using clinical judgement when discontinuing isolation.

**Disclosures:**

**Emily P. Hyle, MD MSc**, UpToDate.com: Royalties **Molly L. Paras, MD**, Angiodynamics: Honoraria|Angiodynamics: Honoraria

